# UBE3A and MCM6 synergistically regulate the proliferation and migration of lung adenocarcinoma cells

**DOI:** 10.1002/2211-5463.13675

**Published:** 2023-07-28

**Authors:** Yanyan Luo, Yun Yang, Cong Yang, Chuanyin Li, Ronggui Hu, Wujun Geng, Xianhui Kang, Hai Lin

**Affiliations:** ^1^ Department of Pain, Wenzhou Key Laboratory of Perioperative Medicine The First Affiliated Hospital of Wenzhou Medical University China; ^2^ State Key Laboratory of Molecular Biology, Shanghai Institute of Biochemistry and Cell Biology Center for Excellence in Molecular Cell Science, Chinese Academy of Sciences Shanghai China; ^3^ School of Medicine Guizhou University Guiyang China; ^4^ Cancer Center, School of Medicine, Shanghai Tenth People's Hospitalf Tongji University Shanghai China; ^5^ Department of Anesthesiology, The First Affiliated Hospital Zhejiang University School of Medicine Hangzhou China

**Keywords:** E6AP, lung adenocarcinoma, MCM6, migration, proliferation

## Abstract

Lung cancer is a leading cause of mortality worldwide and shows substantial clinical and biomolecular heterogeneity. Currently, specific therapeutic strategies are lacking, so effective drug targets are urgently needed. E6AP/UBE3A is a multifaceted ubiquitin ligase that controls various signaling pathways implicated in neurological diseases and various cancers; however, its role in lung cancer is incompletely understood. Here, MCM6 was identified as an interacting partner of E6AP using the yeast two‐hybrid assay. MCM2 and MCM4 were then shown to interact with E6AP. E6AP knockout enhanced the ubiquitination of MCM2/4/6, suggesting that E6AP was not the E3 ubiquitin ligase for these three MCM proteins. Ablation of E6AP inhibited proliferation and migration, but had no significant effect on apoptosis in A549 and H1975 cells, and proliferation and migration inhibition was also observed in MCM6 knockdown cells. Furthermore, ablation of MCM6 and E6AP synergistically suppressed the proliferation and migration of A549 and H1975 cells. To verify the above findings *in vivo*, we established tumor models in nude mice and identified that the tumorigenicity of human lung adenocarcinoma (LUAD) cells was synergistically regulated by MCM6 and E6AP. Moreover, the expression levels of MCM6 and E6AP were higher in LUAD tissues than in adjacent tissues. Furthermore, the expression levels of MCM6 and E6AP were positively correlated in human LUAD samples. Thus, our study suggests that the interaction of E6AP and MCM proteins plays an important role in the progression of LUAD, which might offer potential therapeutic targets for cancer treatment.

## Graphical abstract

E6AP/UBE3A is a multifaceted ubiquitin ligase that controls various signaling pathways. In our study, MCM6, a member of the MCM family, was identified as an interacting partner of E6AP. The interaction plays an important role in the progression of lung adenocarcinoma, which might offer potential therapeutic targets for cancer treatment.

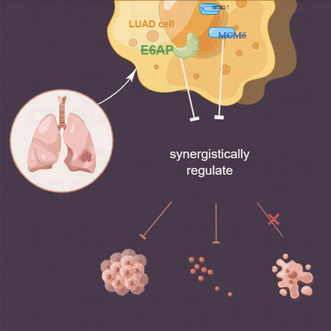

**Abbreviations**
AS: Angelman syndrome
ASD: autism spectrum disorder
CAS: Chinese Academy of Sciences
Co‐IP: co‐immunoprecipitation
E6AP/UBE3A: E6‐associated protein/ubiquitin‐protein ligase E3A
HPV: human papillomavirus
IACUC: Institutional Animal Care and Use Committee
IP: immunoprecipitation
KD: knockdown
KO: knockout
LUAD: lung adenocarcinoma
MCM: minichromosome maintenance proteins
NC: negative control
NSCLC: non‐small‐cell lung cancer
RA: retinoic acid
RT: room temperature
Y2H: *Yeast* two‐hybrid

Lung cancer is a leading cause of mortality worldwide and accounts for 22% of all cancer‐related deaths according to data from the United States [1]. Approximately 85% of lung cancer cases are non‐small‐cell lung cancers (NSCLCs), which mainly include lung adenocarcinoma (LUAD) and lung squamous cell carcinoma [2, 3]. Lung adenocarcinoma is the most common type of lung cancer, and although many other anticancer strategies have been developed for the clinical treatment of LUAD, in addition to surgery, including chemotherapy, immunotherapy, and targeted therapy [4, 5, 6], the long‐term survival rate of patients with LUAD remains low due to tumor recurrence and metastasis. Therefore, it is necessary to understand the molecular mechanism associated with LUAD and identify predictive biomarkers of LUAD recurrence and prognosis to help monitor disease progression and guide treatment.

E6AP, human papillomavirus (HPV) E6‐associated protein, also known as ubiquitin‐protein ligase E3A (UBE3A), is a multifaceted ubiquitin ligase that controls various signaling pathways involved in neurological diseases and cancers [7]. In nervous system diseases, studies have shown that loss‐of‐function mutations in maternal UBE3A account for 8% of Angelman syndrome (AS) cases [8], while hyperactivity of UBE3A accounts for 1–3% of autism spectrum disorder (ASD) cases worldwide [9, 10] and sometimes leads to increased nonproteolytic ubiquitylation of ALDH1A proteins, which compromises retinoic acid (RA) biosynthesis and impairs the activity of neurons [11]. In terms of cancer, in the presence of the HPV E6 protein, E6AP functions as an E3 ligase and degrades the p53 protein, leading to the occurrence of cervical cancer [12]. In esophageal cancer, E6AP activates the NOTCH pathway and promotes tumor progression by degrading ZNF185 [13]. In NSCLC, E6AP deletion enhances the efficiency of immunotherapy [14]. To date, many proteins have been identified as substrates for E6AP, but its role in LUAD has seldom been reported.

MCM6 is one of six members of the minichromosome maintenance family. A previous study verified that MCM6, correlated with HuR, is a valuable marker of poor prognosis in LUAD [15]. The important role of MCM6 in cell proliferation has been reported, indicating its potential function in promoting tumor progression. In this study, minichromosome maintenance (MCM) family proteins were identified as interacting partners for E6AP, and their roles in LUAD were studied. Therefore, E6AP may act as a potential target for LUAD and interact with MCM6 to synergistically regulate the proliferation and migration of LUAD cells.

## Materials and methods

### *Yeast* two‐hybrid assay

*Yeast* two‐hybrid (Y2H) screening was performed as previously described [16] using human E6AP as bait. Positive colonies were picked, subjected to a survival test in SD‐4 (deficient in Ura, His, Leu and Trp) medium, and then stained for β‐glycosidase activity using X‐Gal (Sigma, St.Louis, MO, USA). Identities of the hits were then determined by Sanger sequencing.

### Plasmid construction

The sgRNA targeting E6AP was designed using an online tool (http://crispr.mit.edu/), synthesized as oligos (Qingke, Shanghai, China), annealed, and inserted into a PX330 vector that was digested with *BbsI*, generating PX330‐E6AP‐sgRNA. The specific sequence for sgRNA is shown in Table 1. The shRNAs for MCM6 were synthesized as oligos (Qingke, China), annealed, and inserted into the pLKO.1 vector that was digested with *EcoRI* and *AgeI*, and the specific sequence for shRNA is shown in Table 2. The plasmids of full‐length E6AP and MCM2‐7 were kindly provided by Professor Ronggui Hu (Chinese Academy of Sciences, Shanghai, China). The plasmids of E6AP and MCM6 mutants were generated using the QuikChange Site‐Directed Mutagenesis Kit (Stratagene, San Diego, CA, USA).

**Table 1:** Sequences of the sgRNA primers for E6AP.

sgRNA primers	Sequence (5′–3′)
sg E6AP‐1‐F	TTCCCAATTATCAACAACATGGG
sg E6AP‐1‐R	CAACAGGCACTGATCTGCCATGG
sg E6AP‐2‐F	CACCGATATGACGGTGGCTATACCA
sg E6AP‐2‐R	AAACTGGTATAGCCACCGTCATATC

**Table 2:** Sequences of the shRNA primers for MCM6.

shRNA primers	Sequence (5′–3′)
sh MCM6‐1‐F	CCGGCCCGATTCGATCTCTTCTTTACTCGAGTAAAGAAGAGATCGAATCGGGTTTTTG
sh MCM6‐1‐R	AATTCAAAAACCCGATTCGATCTCTTCTTTACTCGAGTAAAGAAGAGATCGAATCGGG
sh MCM6‐2‐F	CCGGCCTAACTACTTGCTCGAAGATCTCGAGATCTTCGAGCAAGTAGTTAGGTTTTTG
sh MCM6‐2‐R	AATTCAAAAACCTAACTACTTGCTCGAAGATCTCGAGATCTTCGAGCAAGTAGTTAGG

### Cell culture and transfection

The human cervical carcinoma cell line HeLa and the pulmonary carcinoma cell lines A549 and H1975 were purchased from the cell bank of the Chinese Academy of Sciences. The A549 and H1975 cell lines were cultured in 1640 (Corning, Corning, NY, USA) supplemented with 10% fetal bovine serum and 100 μg/L penicillin/streptomycin (Life Technologies, Carlsbad, CA, USA). HEK293T and HeLa cell lines were cultured in DMEM (Corning) supplemented with 10% FBS and 100 μg·L^−1^ penicillin/streptomycin. All these cells were placed in a 37 °C humidified atmosphere of 5% CO_2_. Plasmids were transfected into cells using Lipofectamine 2000 (Life Technologies, USA) according to the manufacturer's instructions, and the stably transfected cell lines were screened by puromycin (2 μg·mL^−1^) for at least 1 week.

### Expression and purification of recombinant proteins

Recombinant proteins were purified as previously described [17]. GST‐E6AP, His6‐MCM6, and their respective truncation proteins and His6‐MCM2/3/4/5/7 were expressed in *BL21 E. coli* cells. After isopropyl‐β‐d‐thiogalactopyranoside (IPTG, Sangon, China) induction, bacterial cells were pelleted, lysed in PBS buffer and incubated with glutathione or Ni2 + TA beads (Sangon, China) to enrich the respective proteins, followed by elution with 300 mm imidazole (Sangon) or 20 mm L‐glutathione reduced (Sangon) dissolved in PBS buffer, and then dialysis in PBS buffer supplemented with 20% glycerol before being aliquoted and preserved at −80 °C.

### GST pull‐down assay

Purified His6 tagged proteins (10 μg): His6‐MCM6 and its truncation proteins, His6‐MCM2/3/4/5/7; GST/GST‐tagged proteins (10 μg): GST‐E6AP and its truncation proteins; and Glutathione Sepharose 4B (Sangon) were incubated at 4 °C overnight in 1000 mL GST pull‐down buffer (20 mm Tris‐Cl, 100 mm NaCl, 5 mm MgCl_2_, 1 mm EDTA, 1 mm DTT, 0.5% NP‐40, 10 mg·mL^−1^ of BSA, pH 8.0). The next day, the beads were pelleted and washed five times with GST pull‐down buffer. The recovered immunoprecipitates were boiled in 50 μL of 2x SDS/PAGE protein loading buffer for 10 min and subjected to immunoblotting analysis.

### Coimmunoprecipitation, immunoprecipitation, and immunoblotting

For coimmunoprecipitation (Co‐IP) assay. In brief, HEK293T cells were cotransfected with E6AP‐HA and empty vector or MCM2/4/6‐Flag plasmids. Forty‐eight hours later, the cells were lysed with 500 μL of co‐IP buffer (50 mm Tris‐Cl, 150 mm NaCl, 5 mm EDTA, 1% NP‐40, pH 7.8) containing a protease inhibitor cocktail (Roche, Basel, Switzerland). Subsequently, the cell lysates were centrifuged and incubated with anti‐Flag affinity gels (Sigma) overnight at 4 °C. For the immunoprecipitation (IP) assay, cells were cotransfected with E6AP‐Myc, HA‐Ub and MCM2/4/6‐Flag plasmids and lysed in 500 μL RIPA buffer (50 mm Tris–HCl, 150 mm NaCl, 5 mm EDTA, 0.1% SDS and 1% NP‐40, pH 7.8) supplemented with a protease inhibitor cocktail. Then, the cell lysates were centrifuged and incubated with anti‐Flag affinity gels overnight at 4 °C. The immunoprecipitates were enriched and denatured at 100 °C for 10 min in 2× SDS/PAGE protein loading buffer. The inputs, immunoprecipitates, and other cell lysates were subjected to 10% SDS/PAGE and transferred to a PVDF membrane (Bio‐Rad, Hercules, CA, USA). The membrane was blocked with 5% skimmed milk at room temperature (RT) for 1 h and incubated with the specified antibodies as follows: for GST Pull‐down assay: anti‐GST (1 : 5000 dilution; HRP‐66001; Proteintech, Chicago, IL, USA), anti‐His (1 : 2000 dilution; 66005‐1‐Ig; Proteintech); for Co‐IP assay: anti‐Flag (1 : 2000 dilution; 80010‐1‐RR; Proteintech), anti‐HA (1 : 1000 dilution; 51064‐2‐AP; Proteintech); for IP assay: anti‐E6AP (1 : 2000 dilution; 10344‐1‐AP; Proteintech), anti‐Flag (1 : 2000 dilution; 80010‐1‐RR; Proteintech), anti‐HA (1 : 1000 dilution; 51064‐2‐AP; Proteintech), anti‐GAPDH (1 : 5000 dilution; 60004‐1‐Ig; Proteintech), for immunoblotting: anti‐E6AP (1 : 2000 dilution; 10344‐1‐AP; Proteintech), anti‐MCM6 (1 : 2000 dilution; 13347‐2‐AP; Proteintech), anti‐E‐cadherin (1 : 5000 dilution; 20874‐1‐AP; Proteintech), anti‐Vinculin (1 : 5000 dilution; 26520‐1‐AP; Proteintech), anti‐CCND1 (1 : 1000 dilution; K00Z196P, Solarbio, Beijing, China), and anti‐Caspase‐3 (1 : 1000 dilution; AC030‐1; Beyotime, Shanghai, China). Then, the membranes were incubated with HRP‐conjugated goat anti‐mouse IgG (1 : 5000 dilution; SA00001‐1; Proteintech) or goat anti‐rabbit IgG (1 : 5000 dilution; SA00001‐2; Proteintech) secondary antibodies, and the signals were visualized using a Tanon 5200 Imaging System (Tanon, Jinan, Shangdong Province, China).

### Immunofluorescence

HeLa cells were cotransfected with plasmids MCM2/4/6‐Flag and E6AP‐PEGFP, fixed with 4% paraformaldehyde 48 h later, treated with 0.5% Triton X‐100 at RT for 10 min, incubated with anti‐Flag antibody (Sigma) at 4 °C overnight, and then incubated with Alexa Fluor 488‐labeled secondary antibody (1 : 5000 dilution; Thermo Fisher, Waltham, MA, USA) and Alexa Fluor 594‐labeled secondary antibody (1 : 5000 dilution; Thermo Fisher) at RT for 1 h. The nuclei of cells were stained with 4′,6‐diamidino‐2‐phenylindole (DAPI, Sigma) for 5 min, and images were taken using an Olympus BX51 microscope (Olympus, Tokyo, Japan).

### Generation of E6AP knockout cell lines

The E6AP knockout (KO) cell line was generated using the CRISPR‐CAS9 technique as previously described [11]. Briefly, A549 and H1975 cells were transfected with CRISPR‐CAS9‐based sgRNA (PX330‐E6AP‐sgRNAs), and monoclonal antibodies were chosen and detected by immunoblotting analysis. Then, genetic ablation of E6AP was confirmed by Sanger sequencing.

### Cell proliferation assay

Cells were seeded into 96‐well plates at a density of 3000 cells/well. Cell Counting Kit‐8 (Yeasen Bio, Shanghai, China) solution was added at different time points (0 h, 24 h, 48 h, and 72 h), and the 0 h time point was defined as 6 h after the cells were seeded. After incubation at 37 °C for 1 h, the absorbance value at 450 nm was measured by a microplate reader (Bio‐Rad). Experiments were conducted with eight replicates and repeated three times.

### Colony formation assay

Cells were seeded into 6‐well plates at a density of 1000 cells/well and cultured for 7 days. The cells were fixed with 4% paraformaldehyde (Yeasen Bio, Shanghai, China) for 15 min at RT, washed twice with PBS, and stained with 0.1% crystal violet (Yeasen Bio, Shanghai, China) for 5 min. Images were obtained by a camera, and the number of colonies was counted and calculated. Experiments were conducted with three replicates and repeated three times.

### Wound‐healing assay

Cells were seeded into 6‐well plates at a density of 10^5^ cells/well. The cells were scraped with a 20‐microliter tip when they grew to 100% confluence. Then, the cells were washed three times with PBS and cultured in serum‐free medium for 24 h. Images were taken using an ECLIPSE Ti2 inverted microscope (Nikon, Tokyo, Japan) every 24 h after scratching. Wound closure was quantified and calculated. All data were obtained from three independent experiments.

### Animal studies

All animal experiments strictly followed the instructions for the care and use of laboratory animals and received the approval of the Institutional Animal Care and Use Committee (IACUC) at the Center for Excellence in Molecular Cell Science, Chinese Academy of Sciences (CAS; 2019‐012). A total of 2 × 10^6^ NC, MCM6 knockdown (KD), E6AP KO and both MCM6 and E6AP KD A549 and H1975 cells mixed with 100 μL of Matrigel (BD Biosciences, Franklin, NJ, USA) were injected into 5‐week‐old male BALB/C nude mice purchased from the Shanghai Laboratory Animal Centre (SLAC, Suzhou, Jiangsu Province, China). The feeding environment requires a temperature ranges from 18 °C to 29 °C, a relative humidity of 40 ~ 70%, and a light cycle of 12 h bright and 12 h dark. The mice were fed with sterile high‐nutrient feed and sterile water, and every six mice were raised in a special cage. After monitoring the tumor sizes for 28 days, the mice were killed, and the tumors were surgically removed and weighed.

### Immunohistochemistry

Human LUAD tissue arrays were purchased from Shanghai Servicebio. Immunohistochemistry for tissues from 79 patients with lung cancer was performed as previously reported. Briefly, tissues were fixed in formalin, embedded in paraffin, and sectioned before being mounted on slides, which were then subjected to deparaffinization and rehydration. Then, the slides were microwaved for 30 min in 0.01 mol·L^−1^ sodium citrate buffer (pH 6.0). After antigen retrieval and preincubation with 10% normal goat serum, anti‐E6AP (1 : 100 dilution; 10344‐1‐AP; Proteintech) and anti‐MCM6 (1 : 100 dilution; 13347‐2‐AP; Proteintech) were used at 4 °C overnight. These slides were stained by means of the VECTSDTSIN Elite ABC Kit (Vector Laboratories, Burlingame Station, CA, USA) and counterstained with hematoxylin. The density of positive staining was measured using a computerized image system (Leica Microsystems Imaging Solutions, Wetzlar, Hessen, Germany). Photographs of three representative fields were captured using Leica win plus v3 software under high‐power magnification (100×), and identical settings were used for each photograph. The density was counted using image‐pro plus 6.0 software (Media Cybernetics, Silver Springs, MD, USA). A uniform setting for all slides was applied for the reading of each antibody staining. The integrated optical density of all the positive staining in each photograph was measured, and its ratio to the total area of each photograph was calculated as the density.

### Statistical analysis

The data were analyzed with one‐way ANOVA with Tukey's *post hoc* test using graphpad prism 5 (GraphPad Software Inc., San Diego, CA, USA). *P* < 0.05 was considered to indicate a significant difference, and single, double, triple, and quadruple asterisks indicate statistical significance of *P < 0.05, **P < 0.01, ***P < 0.001, ****P < 0.0001.

## Results

### E6AP interacts with the MCM family member MCM6

**Fig. 1:**
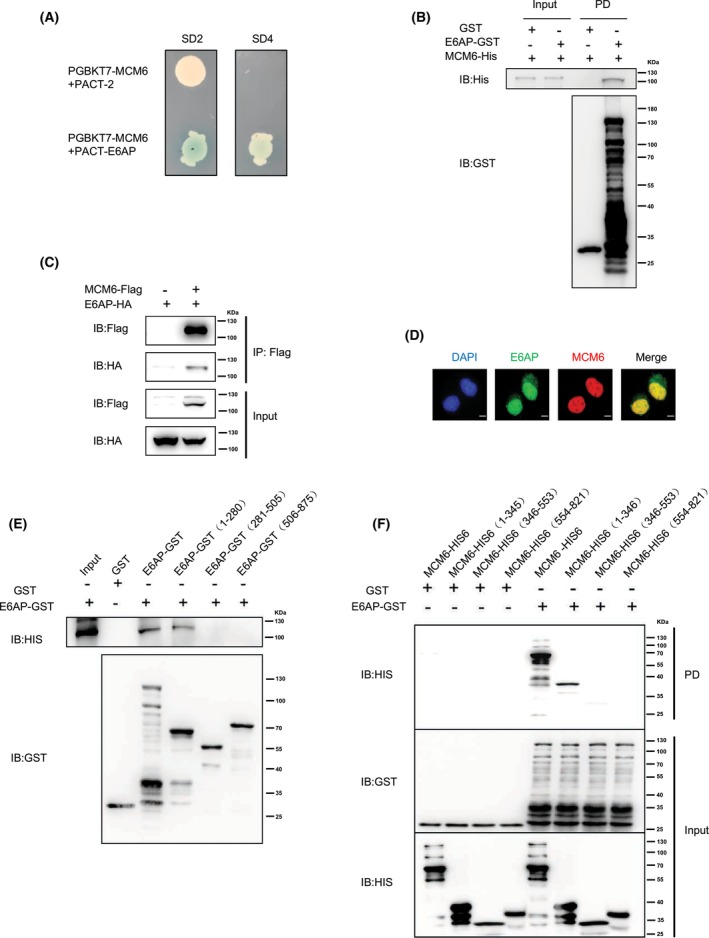
MCM family member MCM6 interacts with E6AP. (A) MCM6 was identified as an interacting partner of E6AP. E6AP was used as bait. SD‐2, deficient in Leu and Trp; SD‐4, deficient in Ura, His, Leu, and Trp. (B) E6AP directly interacted with MCM6 *in vitro*. Recombinant GST‐tagged E6AP and His6‐tagged MCM6 were purified and subjected to a GST pull‐down assay. (C) Exogenously expressed E6AP and MCM6 formed a complex. 293T cells were cotransfected with Flag‐tagged MCM6 and HA‐tagged E6AP, and the cell lysates were centrifuged, incubated with anti‐Flag affinity gels, and then subjected to a Co‐IP assay. (D) GFP‐tagged E6AP colocalized with MCM6. HeLa cells were cotransfected with GFP‐tagged E6AP and Flag‐tagged MCM6 and subjected to an immunofluorescence assay. Then, the cells were incubated with anti‐Flag antibody and Alexa Fluor secondary antibody. The nuclei of cells were stained with 4′,6‐diamidino‐2‐phenylindole (DAPI). Scale bar: 10 μm. (E, F) The N terminus of E6AP interacted with the N terminus of MCM6. GST pull‐down assays were performed with recombinant GST‐E6AP or its fragments and MCM6‐His6 or its fragments. PD, pull‐down.

To identify the proteins interacting with E6AP, Y2H assays were employed to screen for E6AP‐interacting partners. In total, 27 colonies survived on the SD‐4 plates, and plasmids recovered from these colonies were subjected to individual Sanger sequencing, including MCM6, ALDH1A2, RAD23A, PSMD4, SERPINB2, MSTO1, IL24, and CRP. Among them, 17 clones contained MCM6, so they were selected for further study. MCM6, a member of the MCM complex, was identified as an interacting partner of E6AP and validated in *yeast* cells (Fig. 1A). GST pull‐down showed that E6AP directly interacted with MCM6 *in vitro* and a Co‐IP assay indicated that they formed a complex in 293T cells (Fig. 1B,C). E6AP colocalized with MCM6 in the nucleus of HeLa cells, as revealed by immunofluorescence assay (Fig. 1D). Further GST pull‐down assays demonstrated that the N‐terminal region of E6AP (1‐280) interacted with the N terminus of MCM6 (1‐345) (Fig. 1E,F).

### E6AP interacts with the MCM family member MCM2/4

**Fig. 2:**
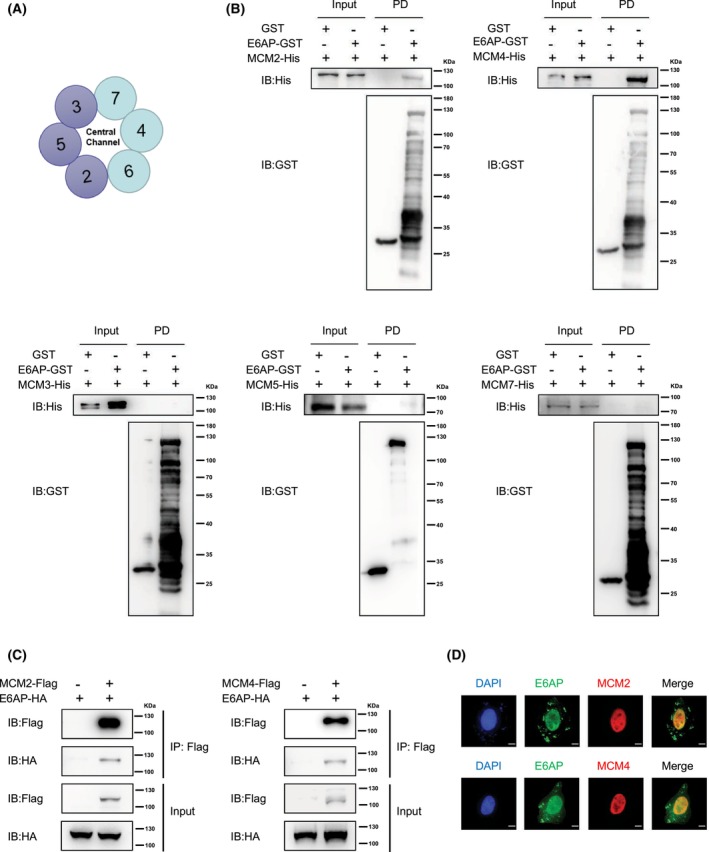
MCM family members MCM2/4 interact with E6AP. (A) Schematic diagram of the MCM2‐7 complex. (B) MCM2/4 directly interacted with E6AP *in vitro*. GST pull‐down assays were performed with recombinant GST‐E6AP and His6‐tagged MCM2/3/4/5/7. PD, pull‐down.(C) Exogenously expressed E6AP and MCM2/4 formed a complex. 293T cells were cotransfected with Flag‐tagged MCM2 or MCM4 and HA‐tagged E6AP, and the cell lysates were centrifuged and incubated with anti‐Flag affinity gels and then subjected to a Co‐IP assay. (D) GFP‐tagged E6AP colocalized with MCM2/4. HeLa cells were cotransfected with GFP‐tagged E6AP and Flag‐tagged MCM2 or MCM4 and subjected to an immunofluorescence assay. Then, the cells were incubated with anti‐Flag antibody and Alexa Fluor secondary antibody. The nuclei of cells were stained with 4′,6‐diamidino‐2‐phenylindole (DAPI). Scale bar: 10 μm.

MCM6 is a member of the MCM complex and forms the MCM2‐7 complex together with MCM2, MCM3, MCM4, MCM5, and MCM7 (Fig. 2A). Then, the interaction of E6AP with other members of the MCM2‐7 complex was tested. As shown in Fig. 2B, GST pull‐down assays showed that E6AP only directly interacted with MCM2 and MCM4 but not with MCM3, MCM5, and MCM7. The Co‐IP assay showed that MCM2 and MCM4 could form a complex with E6AP in 293T cells (Fig. 2C). In addition, immunofluorescence assays showed that MCM2 and MCM4 colocalized with E6AP in the nucleus of HeLa cells (Fig. 2D).

### Ablation of E6AP enhances the ubiquitination of MCM2/4/6

**Fig. 3:**
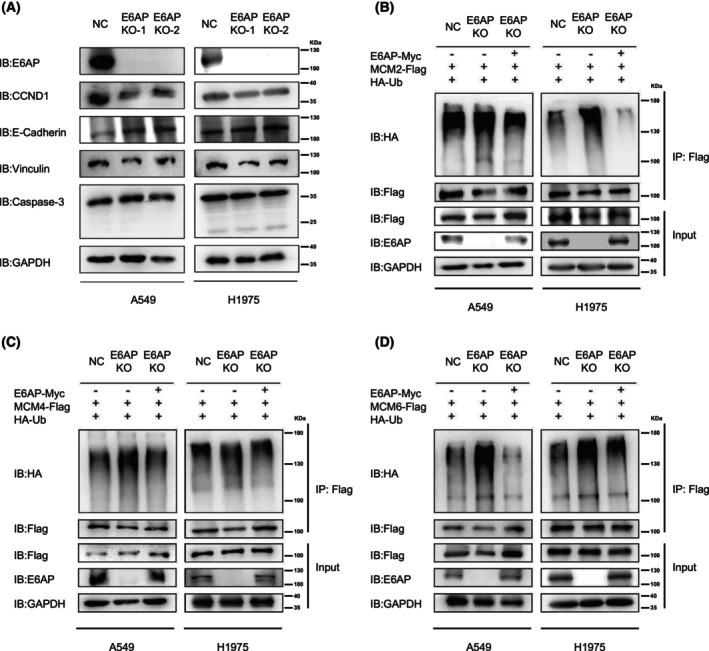
Ablation of E6AP enhances the ubiquitination of MCM2/4/6. (A) E6AP knockout (KO) cell lines were generated using the CRISPR‐CAS9 gene editing approach, and proliferation‐, migration‐, and apoptosis‐related molecular markers were detected by immunoblotting. CCND1, cell proliferation‐related molecule; E‐cadherin and Vinculin, cell migration‐related markers, Caspase‐3, apoptosis‐related molecule. NC, negative control. (B) The ablation of E6AP enhanced the ubiquitination of MCM2. E6AP knockout or NC A549 and H1975 cells were cotransfected with HA‐Ub, MCM2‐Flag, and E6AP‐Myc as indicated. The lysates of these cells were immunoprecipitated with anti‐Flag affinity gels and subjected to immunoblotting analysis. (C) The ablation of E6AP enhanced the ubiquitination of MCM4. E6AP knockout or NC A549 and H1975 cells were cotransfected with HA‐Ub, MCM4‐Flag and E6AP‐Myc as indicated. The lysates of these cells were immunoprecipitated with anti‐Flag affinity gels and subjected to immunoblotting analysis. (D) The ablation of E6AP enhanced the ubiquitination of MCM6. E6AP knockout or NC A549 and H1975 cells were cotransfected with HA‐Ub, MCM6‐Flag and E6AP‐Myc as indicated. The lysates of these cells were immunoprecipitated with anti‐Flag affinity gels and subjected to immunoblotting analysis.

To explore the effect of E6AP on MCM proteins, two E6AP KO A549 and H1975 cell lines were generated using the CRISPR‐CAS9 technique. Immunoblotting analysis showed that the cell proliferation marker CCND1 was obviously downregulated in E6AP KO cells compared with negative control (NC) cells. The cell migration‐related molecule (Vinculin) was obviously downregulated, while the cell migration‐related molecule E‐cadherin was upregulated in LUAD cells with E6AP KO compared with the NC group. In addition, there was no significant difference in the apoptosis‐related molecule Caspase‐3 between the E6AP KO and control groups (Fig. 3A). Then, we tested whether E6AP is an E3 ligase for MCM2/4/6. As shown in Fig. 3B–D, compared with NC cells, the ubiquitination of MCM2/4/6 was increased in E6AP KO LUAD cells, and this effect was reversed when E6AP was reintroduced into E6AP^−/−^ cells (Fig. 3B–D). The results suggested that E6AP was not an E3 ligase for MCM2/4/6.

### E6AP ablation inhibits the proliferation and migration of LUAD cells

**Fig. 4:**
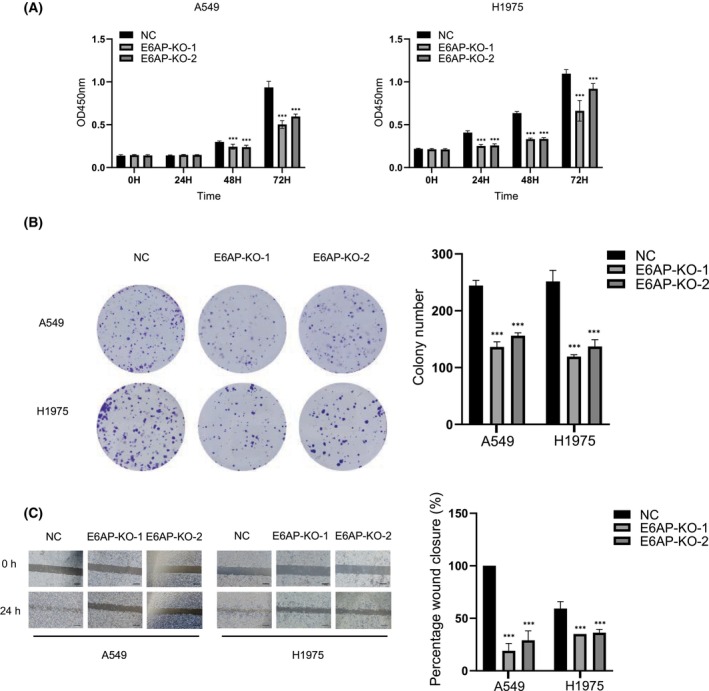
E6AP ablation inhibits the proliferation and migration of LUAD cells. (A) Ablation of E6AP inhibited the proliferation of LUAD cells. The viability of NC and E6AP KO A549 and H1975 cells was detected by the CCK‐8 assay at different time points (0, 24, 48, and 72 h). The 0 h time point was defined as 6 h after the cells were seeded into 96‐well plates. The data are expressed as the mean ± SD and were analyzed using one‐way ANOVA with Tukey's *post hoc* test. ***P < 0.001, very significantly different, three independent experiments. (B) Ablation of E6AP inhibited the colony formation of LUAD cells. NC or E6AP KO A549 and H1975 cells were seeded into 6‐well plates at 1000 cells per well and cultured for 7 days. The colonies were fixed and stained, and images were acquired using a camera. The colony numbers were counted and calculated. The data were expressed as the mean ± SD and analyzed using one‐way ANOVA with Tukey's *post hoc* test. ***P < 0.001, very significantly different. three samples for each group and three independent experiments. (C) Ablation of E6AP inhibited the migration of LUAD cells. NC and E6AP KO A549 and H1975 cells were seeded into 6‐well plates, and a wound‐healing assay was performed. Wound closure was quantified and calculated. ***P < 0.001, very significantly different. All data were obtained from three independent experiments. Scale bar: 500 μm. KO, knockout; NC, negative control.

Phenotypic experiments were performed to explore the effect of E6AP on LUAD cells. First, cell viability was detected by CCK‐8 assay at different time points (0, 24, 48, and 72 h), and obvious cell growth arrest was observed in E6AP KO cell lines compared with scramble A549 and H1975 cells (Fig. 4A). Next, a colony formation assay was performed, and decreased colony numbers were found in the E6AP‐KO groups compared with the control group (Fig. 4B). Then, the wound‐healing assay indicated that E6AP‐KO cells maintained longer widths 24 h after scratching than NC cells, which exhibited much smaller widths. All these data indicated that KO of E6AP markedly suppressed cell proliferation and migration in LUAD cells.

### MCM6 KD inhibits the proliferation and migration of LUAD cells

**Fig. 5:**
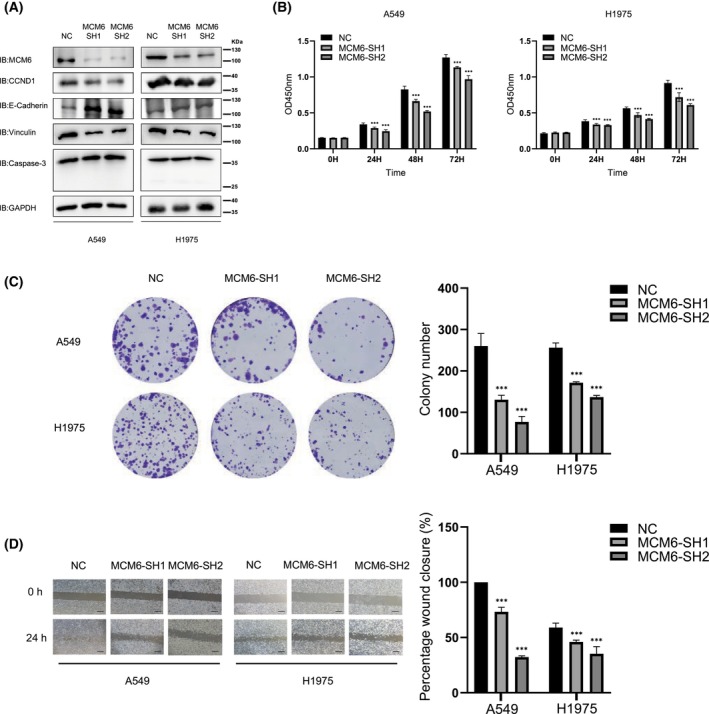
MCM6 KD inhibits LUAD cell proliferation and migration. (A) Test of the KD efficiency of shRNAs for MCM6. A549 and H1975 cells that stably expressed MCM6 shRNA were subjected to immunoblotting analysis. The detection of proliferation‐, migration‐ and apoptosis‐related molecular markers was also tested. CCND1, cell proliferation‐related molecule; E‐cadherin and Vinculin, cell migration‐related markers, Caspase‐3, apoptosis‐related molecule. (B) MCM6 KD inhibits the proliferation of LUAD cells. The viability of A549 and H1975 cells that were stably transfected with MCM6 shRNAs was detected by the CCK‐8 assay at different time points (0, 24, 48, and 72 h). The data are expressed as the mean ± SD and were analyzed using one‐way ANOVA with Tukey's *post hoc* test. ***P < 0.001, very significantly different. All data were obtained from three independent experiments. (C) MCM6 KD inhibited the colony formation of LUAD cells. A549 and H1975 cells that were stably transfected with MCM6 shRNAs were seeded into 6‐well plates at 1000 cells per well and cultured for 7 days. The colonies were fixed and stained, and images were acquired using a camera. The colony numbers were counted and calculated. The data were expressed as the mean ± SD and analyzed using one‐way ANOVA with Tukey's *post hoc* test. ***P < 0.001, very significantly different. All data were obtained from three independent experiments. (D) MCM6 KD inhibited the migration of LUAD cells. A549 and H1975 cells that stably expressed MCM6 shRNAs were seeded into 6‐well plates, and a wound‐healing assay was performed. Wound closure was quantified and calculated. ***P < 0.001, very significantly different. Scale bar: 500 μm. All data were obtained from three independent experiments. NC, negative control.

To investigate the role of MCM6, two shRNAs targeting MCM6 were designed and tested in LUAD cells. Immunoblotting analysis indicated that cells transfected with sh1 and sh2 showed little MCM6 protein levels compared with the NC group (Fig. 5A). Immunoblotting analysis showed that the cell proliferation marker CCND1 was obviously downregulated in MCM6 KD cells compared with NC cells. We also found that in MCM6 KD cell lines, the expression level of the cell migration‐related molecule Vinculin decreased, while the cell migration‐related molecule E‐cadherin showed the opposite trend. Moreover, the expression of the apoptosis‐related molecule Caspase‐3 was not obviously different. The viability of LUAD cells was decreased in the MCM6 KD groups compared with the NC group, as revealed by the CCK‐8 assay (Fig. 5B). Furthermore, a colony formation assay was performed, and colony numbers were calculated. Decreased colony numbers were observed in MCM6 KD cells compared with cells in the NC group (Fig. 5C). Wound‐healing assays indicated that MCM6 KD cells maintained longer widths 24 h after scratching than LUAD cells in the NC group, which exhibited much smaller widths (Fig. 5D). These data suggested that MCM6 KD inhibits the proliferation, migration, and colony formation of LUAD cells.

### Ablation of UBE3A and MCM6 synergistically suppresses the proliferation and migration of LUAD cells

**Fig. 6:**
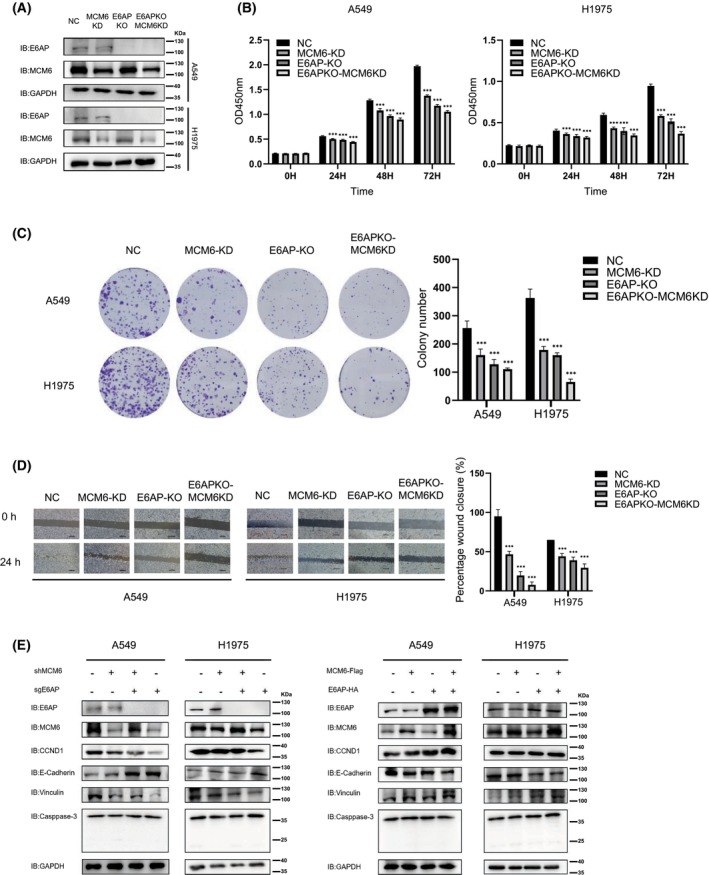
UBE3A and MCM6 synergistically regulate the proliferation and migration of LUAD cells. Detection of the protein levels of E6AP and MCM6 in LUAD cells with different genetic states. E6AP knockout A549 and H1975 cells were stably transfected with MCM6 shRNA, and the expression of E6AP and MCM6 was detected by immunoblotting analysis. UBE3A and MCM6 synergistically inhibited the proliferation of LUAD cells. The viability of A549 and H1975 cells with E6AP knockout and/or MCM6 knockdown was detected by the CCK‐8 assay at different time points (0, 24, 48, and 72 h). The data are expressed as the mean ± SD and were analyzed using one‐way ANOVA with Tukey's *post hoc* test. ***P < 0.001, very significantly different. All data were obtained from three independent experiments. UBE3A and MCM6 synergistically inhibited the colony formation of LUAD cells. A549 and H1975 cells with E6AP knockout and/or MCM6 knockdown were seeded into 6‐well plates at 1000 cells per well and cultured for 7 days. The colonies were fixed and stained, and images were acquired using a camera. The colony numbers were counted and calculated. The data are expressed as the mean ± SD and were analyzed using one‐way ANOVA with Tukey's *post hoc* test. ***P < 0.001, very significantly different. All data were obtained from three independent experiments. (D) UBE3A and MCM6 synergistically inhibited the migration of LUAD cells. A549 and H1975 cells with E6AP knockout and/or MCM6 knockdown were seeded into 6‐well plates, and a wound‐healing assay was performed. Wound closure was quantified and calculated. ***P < 0.001, very significantly different. Scale bar: 500 μm. All data were obtained from three independent experiments. (E) Detection of proliferation‐, migration‐, and apoptosis‐related molecular markers. The lysates of A549 and H1975 cells with E6AP knockout and/or MCM6 knockdown were subjected to immunoblotting analysis. CCND1, cell proliferation‐related molecule; E‐cadherin and Vinculin, cell migration‐related markers, Caspase‐3, apoptosis‐related molecule. KD, knockdown; KO, knockout; NC, negative control.

E6AP KO LUAD cells were stably transfected with shRNA targeting MCM6, and the expression of E6AP and MCM6 in cells with different genetic states was detected by immunoblotting analysis (Fig. 6A). Compared with LUAD cells with E6AP KO or MCM6 KD, decreased cell viability and colony numbers were observed in cells with both E6AP KO and MCM6 KD (Fig. 6B,C). The migration ability of LUAD cells with E6AP KO and MCM6 KD was decreased compared with that of cells in other groups, as revealed by the wound‐healing assay (Fig. 6D). Then, proliferation‐, migration‐, and apoptosis‐related molecular markers were detected in these two cell lines with different genetic states. As shown in Fig. 6E, immunoblotting analysis indicated that the cell proliferation marker CCND1 and cell migration‐related molecule (Vinculin) were obviously downregulated in A549 and H1975 cells with E6AP KO and/or MCM6 KD compared with the control group, while the cell migration‐related molecule E‐cadherin was upregulated in A549 and H1975 cells with E6AP KO and/or MCM6 KD compared with the control group. The expression of the apoptosis‐related molecule Caspase‐3 was not obviously different. These results suggested that the ablation of UBE3A and MCM6 synergistically suppresses the proliferation and migration of LUAD cells.

### Ablation of E6AP and MCM6 synergistically suppresses the tumorigenicity of LUAD cells in nude mice

**Fig. 7:**
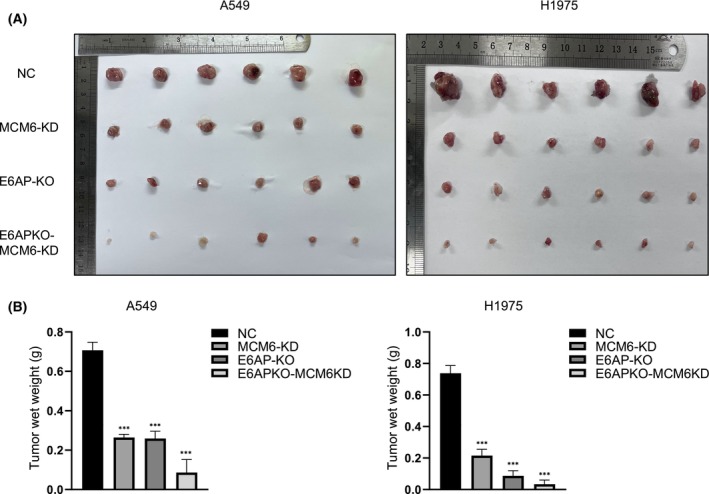
Ablation of E6AP and MCM6 synergistically suppresses the tumorigenicity of LUAD cells in nude mice. (A) Images show xenograft tumors of nude mice 28 days after injection. (B) The weights of the tumors were measured and calculated. The data are expressed as the mean ± SD and were analyzed using one‐way ANOVA with Tukey's *post hoc* test. Six samples were used for each group. ***P < 0.001, very significantly different. All data were obtained from three independent experiments. KD, knockdown; KO, knockout; NC, negative control.

A tumorigenicity model in nude mice was employed to further probe the effects of E6AP and MCM6 on A549 and H1975 cells. LUAD cells with different genetic states were injected into nude mice, and the mice were sacrificed at Day 28 postinjection. The tumors were removed from the mice, and the weights of the tumors were measured and calculated. Compared with the NC group, smaller tumors were observed in the E6AP KO or MCM6 KD group, and mice in the group with both E6AP KO and MCM6 KD showed even smaller tumors than those in the other groups (Fig. 7A,B). Thus, this study suggests that MCM6 and E6AP synergistically suppress the proliferation of LUAD cells and their xenograft tumors in nude mice.

### E6AP and MCM6 are highly expressed in patients with LUAD and are positively correlated

**Fig. 8:**
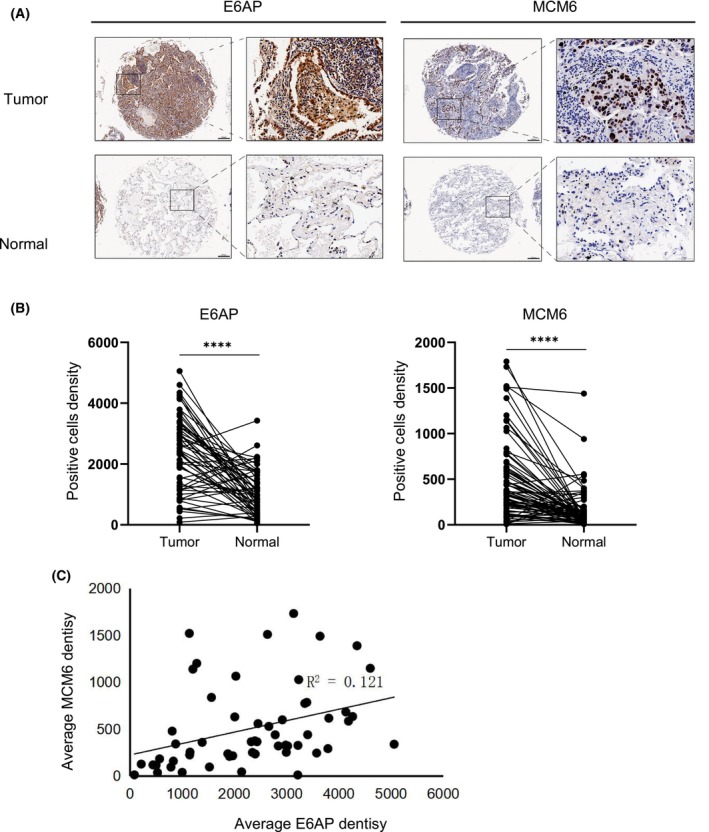
E6AP and MCM6 are highly expressed in patients with LUAD and are positively correlated. Representative immunohistochemistry of MCM6 and E6AP was detected in human LUAD or adjacent tissues. The tumor and adjacent tissues of LUAD patients were sectioned and immunostained with anti‐E6AP and MCM6 antibodies, respectively. Scale bar: 200 μm (single case chip overall picture). The density of E6AP and MCM6 in human LUAD and adjacent tissues. Each LUAD sample and its corresponding adjacent tissues are connected by solid lines, ****P < 0.0001, the difference was very significant. The correlation between E6AP and MCM6 expression in human LUAD.

Tissues from patients with human LUAD were immunostained with anti‐E6AP or anti‐MCM6 antibodies. The expression levels of E6AP and MCM6 in cancer tissues were both higher than those in adjacent tissues (Fig. 8A,B). Correlation analysis showed that the expression of E6AP and MCM6 in human LUAD samples was significantly positively correlated (Fig. 8C). This result indicates that E6AP and MCM6 have synergistic effects to some extent, which is consistent with the previous results.

## Discussion

E6AP/UBE3A is involved in many diseases, including human cancers, and a variety of proteins have been identified as its substrates, including p53, TSC2, RAD23A, ALDH1A2, Smurf1, and GRIM‐19 [7, 11, 18, 19, 20]. For example, SMURF1 has been shown to act as a tumor promoter through ubiquitination modification and/or degradation of tumor suppressor proteins [21]. GRIM‐19 was reported to play an important role in inducing the accumulation of p53 and inhibiting the proliferation and metastasis of cervical cancer cells [22]. In this study, MCM6 was first identified as an interacting partner of E6AP using the Y2H method and then validated by GST pull‐down and Co‐IP assays (Fig. 1). Then, the interaction of E6AP with other members of the MCM2‐7 complex was tested by GST pull‐down assay, and only the interaction between E6AP and MCM2 and MCM4 was detected, but not with MCM3, MCM5, and MCM7 (Fig. 2B). Contrary to our results, MCM7 was identified as a substrate for E6AP [23], and the reason needs to be further explored.

Surprisingly, the ablation of E6AP enhanced the ubiquitination of MCM2/4/6 (Fig. 3), which means that MCM2, MCM4, and MCM6 are not substrates for the E3 ligase E6AP. A possible explanation is that E6AP competes with E3 ligase for interaction with MCMs, which leads to reduced ubiquitination of MCM2/4/6 mediated by E3 ligase. We tried to identify the E3 ligase for MCM6 using Y2H methods but failed. In the future, multiple methods will be combined to identify the E3 ligase for MCM6.

The function of E6AP in lung cancer has seldom been reported. Gamell et al. [24] found that reduced abundance of the E3 ubiquitin ligase E6AP contributes to decreased expression of the INK4/ARF locus in NSCLC, which suggests that E6AP acts as a tumor suppressor. However, our data showed that E6AP KO inhibited the proliferation and migration of A549 and H1975 cells (Fig. 4A–C). This may be due to the different cell lines used in these two studies. Whether E6AP acts as a tumor suppressor gene or an oncogene may be related to the type of cell line.

The minichromosome maintenance (MCM) proteins are essential replication initiation factors originally identified as proteins required for minichromosome maintenance in Saccharomyces cerevisiae [25, 26]. The MCM family consists of 10 proteins, and MCM 2‐9 are characterized by the presence of the ATPase domain (MCM box); MCM 1 and MCM 10 are involved in DNA replication but do not have a functional domain [27]. The best‐known six structurally related proteins, MCM2‐7, are evolutionally conserved in all eukaryotes. Together with CDC6 and CDT1, MCMs act as components of the prereplicative complex. Additional factors, such as cyclin‐dependent kinase (CDK) and polymerase, are recruited to activate DNA unwinding and initiate DNA replication during S phase [28, 29]. MCMs are markers for proliferation, and abnormalities in their function result in chromosomal defects that contribute to tumorigenesis [28]. The expression of MCM6 is positively correlated with cell proliferation, migration, invasion, and the immune response in many cancer types, such as breast cancer and lung cancer [30]. Consistent with other research, MCM6 KD inhibited the proliferation and migration of LUAD cells (Fig. 6A–C).

Further study demonstrated that the ablation of MCM6 and E6AP synergistically suppressed the proliferation, migration and tumorigenicity of LUAD cells. The expression of E6AP and MCM6 in LUAD tissues was higher than that in adjacent tissues and showed a positive correlation. Thus, our study suggests that the interaction of E6AP and MCMs plays an important role in the progression of lung cancer, which might offer potential therapeutic targets for cancer treatment. The changes in E6AP and MCM6 at the molecular level and phenotype of lung cancer can be used to direct the early diagnosis of an active intervention in LUAD patients.

## Conflict of interest

The authors declare no conflict of interest.

### Peer review

The peer review history for this article is available at https://www.webofscience.com/api/gateway/wos/peer‐review/10.1002/2211‐5463.13675.

### Author contributions

XK, HL, RH, and WG contributed to conception. YL, YY, and CY contributed to interpretation or analysis of data and preparation of the manuscript. RH, XK, HL, and WG contributed to supervision.

## Data Availability

All the data generated or analyzed during this study are included in the published article, and the cell lines and plasmids could be offered on reasonable request.
